# Dermatoscopic features of pyogenic granuloma in children

**DOI:** 10.1002/pdi3.73

**Published:** 2024-06-17

**Authors:** Shijuan Yu, Hua Wang, Qi Tan, Li Wang, Xiaoyan Luo, Jingyi He

**Affiliations:** ^1^ Department of Pediatric Dermatology Children's Hospital of Chongqing Medical University Chongqing China; ^2^ Ministry of Education Key Laboratory of Child Development and Disorders Chongqing China; ^3^ National Clinical Research Center for Child Health and Disorders Chongqing China; ^4^ China International Science and Technology Cooperation Base of Child Development and Critical Disorders Chongqing China

**Keywords:** analysis, dermatoscopy, features, pyogenic granuloma

## Abstract

The clinical appearances of Pyogenic granuloma (PG) in children are not as typical as in adults. Meanwhile, skin biopsy is hard to accept for parents in most of time. Therefore, data of PG in children has been deprived. To analyze the dermoscopic features of PG in children in order to improve the efficiency of diagnosis and treatment and to reduce the probability of invasive examination. A single‐center retrospective study conducted from 1 January 2022 to 30 August 2022 summarized and analyzed the clinical data and dermoscopic features of patients clinically diagnosed with PG. A total of 36 patients were involved, 61.11% were males, 72.22% occurred in the face. Over 50% patients had the following dermoscopic patterns: reddish homogeneous area (100%), white collarette (91.7%), yellow‐white scales (69.4%), vascular structures (52.8%), and white rail lines (55.6%). Regression analysis found that gender and courses of disease had a statistically significant impact on part of the dermoscopic patterns. The proportions of dermoscopic features with yellow‐white scales (69.4%), dark red scabs (27.8%), and bleeding (27.8%) in this study were higher than in previous studies (26.7%, 10%, and 10%, respectively). Dermoscope is a practical diagnostic tool for PG in children. It is necessary to consider gender, disease course and lesion locations when PG is diagnosed using dermoscope. The characteristics of yellow‐white scales, dark red scabs, and bleeding are the microscopic features that distinguish pediatric patients with PG from adult patients, which should be given special attention to in pediatric patients.

## Graphical abstract

Some dermoscopic patterns could help clinicians to diagnose pyogenic granuloma in pediatric patients more efficiently and more accurately. We found that the composed dermoscopic pattern of reddish homogeneous area, white rail lines, and white collarette sign have the highest sensitivity to the diagnosis of pyogenic granuloma and specificity.

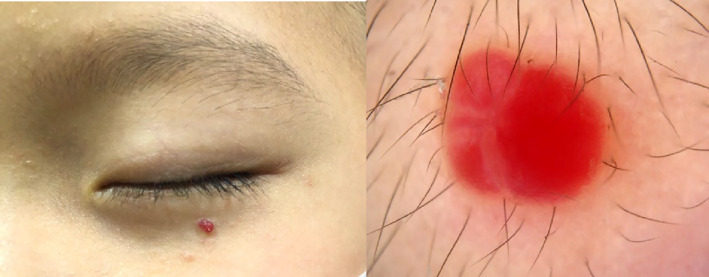

## 1 INTRODUCTION

Pyogenic granuloma (PG) is a common benign vascular tumor,^1^ which is characterized by reddish, soft, fragile and easily‐bleeding papules or nodules, and is usually related to inflammation, trauma, hormones or drugs.^2^, ^3^ PG mainly occurs in the exposed or vulnerable parts of the body such as face, hands, feet, oral mucosa, etc. Up to 1/3 of the patients have a history of trauma before the onset of the disease, especially in the patients with lip injuries or in pediatric patients.^4^, ^5^ The disease could be diagnosed through its history and clinical manifestations, but it needs to be differentiated from benign and malignant skin tumors such as amelanotic malignant melanoma, Spits nevus, infant hemangioma, etc. Histopathological examination is often required, especially for adult patients with increased incidence rate of malignant diseases, to exclude possible malignant skin tumors.^6^ However, invasive histopathological examination is hard to be accepted by pediatric patients and their families. While PG has early onset age for pediatric patients and early clinical manifestations are often not typical. Moreover, children patients and their families often have inaccurate descriptions of the medical history, which might increase the probability of misdiagnosis or missed diagnosis. According to a survey, 38% diagnosis of PG has been proved to be inaccurate only based on clinical manifestations of patients.^7^ Dermatoscopy, as a simple and non‐invasive inspection technique, is able to clearly find the characteristics of superficial vascular diseases of the skin based on the principle of optical amplification. Under the dermoscope, PG can appear as reddish homogeneous areas, white rail lines, white collarette signs at the edge of the skin lesions, yellow‐white scales on the surface of the skin lesions, dark red blood scabs, vascular structures with large, tortuous and dilated vessels in or around the skin lesions.^8^ Dermatoscopy is used for auxiliary diagnosis of PG, which could improve the accuracy and efficiency of diagnosis by avoiding unnecessary invasive examination, and can provide basis and guidance for early treatment to PG.^9^ The dermoscopic patterns of PG have been described in previous studies.^7^, ^10^ However, the dermoscopic patterns described in these studies are limited and general, and there is a lack of data for pediatric population or early stage of the disease. Therefore, this article summarized and analyzed the dermoscopic patterns of PG in pediatric patients, in order to improve the understanding of the dermoscopic patterns of early PG, to provide reference for the early diagnosis and evaluation of PG, to reduce the probability of invasive examination to children patients, and to improve the efficiency of diagnosis and treatment.

## 2 MATERIALS AND METHODS

Retrospective data of dermatoscopy (Guangzhou Chuanghong Medical Technology Co., Ltd., medical electronic dermoscope imaging system, specification and model: CH‐DSIS‐2000 Plus, optical lens magnification: 50X) patterns of 0–18 year‐old children with clinical diagnosis of PG from January 2022 to August 2022 in our hospital(including 7 dermoscopic patterns: reddish homogeneous areas, white rail lines, white collarette signs at the edge of the skin lesions, yellow‐white scales on the surface of the skin lesions, dark red blood scabs, bleeding, vascular structures with large, tortuous and dilated vessels in or around the skin lesions) and clinical data(age, gender, course of disease, location) were collected. We retrospectively analyzed the skin biopsy results of 4 patients, who subsequently diagnosed with PG histopathologically. The study protocol was approved by the Local Research Ethics Committee of the Children's Hospital of Chongqing Medical University, Chongqing, China. The oral informed consents of the child and his family members were obtained.

The continuous data is represented by mean ± standard deviation, and the classified data is represented by numbers (N) and percentages (%). The quantitative data of non‐normal distribution are described and statistically with the median percentile M (P25, P75). Chi‐square test was used to compare the classification variables. Non‐parametric rank sum test (Mann‐Whitney test) was used to compare the differences in the location, age and course of the disease in patients of different sexes. Multivariate logistic regression was used to analyze the causal relationship between the gender, age, course of disease, location of patients and the secondary outcome variables of various dermoscopic characteristics. Multivariate linear regression was used to analyze the correlation between sex, age, disease course and location and the number of dermatoscopic features. All analyses were performed using the Social Science Statistics Software Package (SPSS), version 26.0 (IBM, Amonk, New York, USA). In all analyses, bilateral *p* value < 0.05 was considered statistically significant.

## 3 RESULTS

**FIGURE 1:**
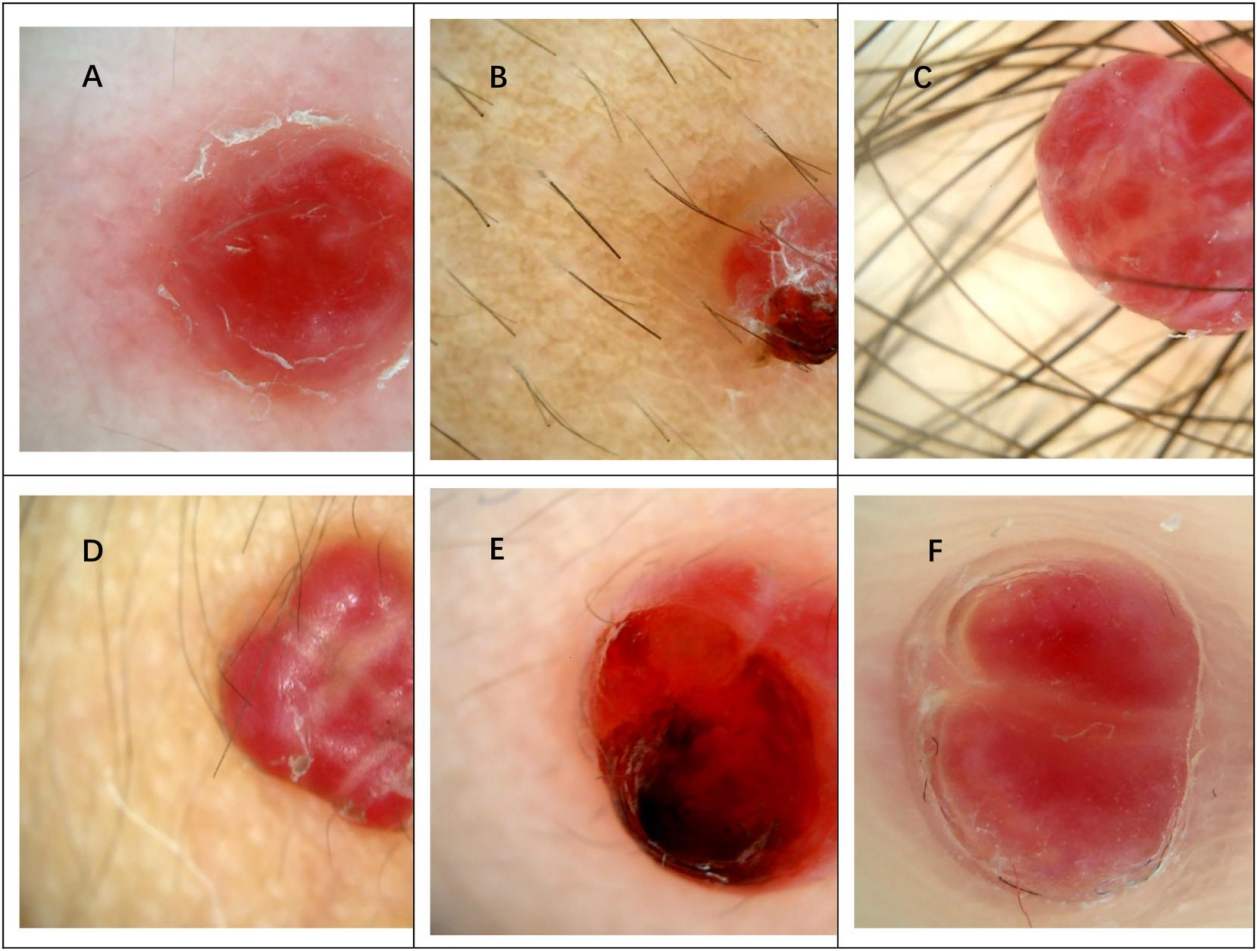
(A) (on the Lower limb) Pattern composed of the presence of reddish or red and white homogeneous area, white collarette, off white scales and linear‐irregular vascular structures. (B) (on the back neck) Pattern composed of the presence of reddish or red and white homogeneous area, white collarette, off white scales, hemorrhage and dark red scabs. (C) (on the face) Pattern composed of the presence of reddish or red and white homogeneous area, white rail lines and off white scales. (D) (on the neck) Pattern composed of the presence of reddish homogeneous area, white collarette, Brownish rail lines and off white scales. (E) (On the face) Pattern composed of the presence of reddish homogeneous area, white collarette, hemorrhage, dark red scabs, linear‐irregular vascular structures and white rail lines. (F) (on the Lower limb) Pattern composed of the presence of reddish or red and white homogeneous area, white collarette, white rail lines and vascular structures.

**FIGURE 2:**
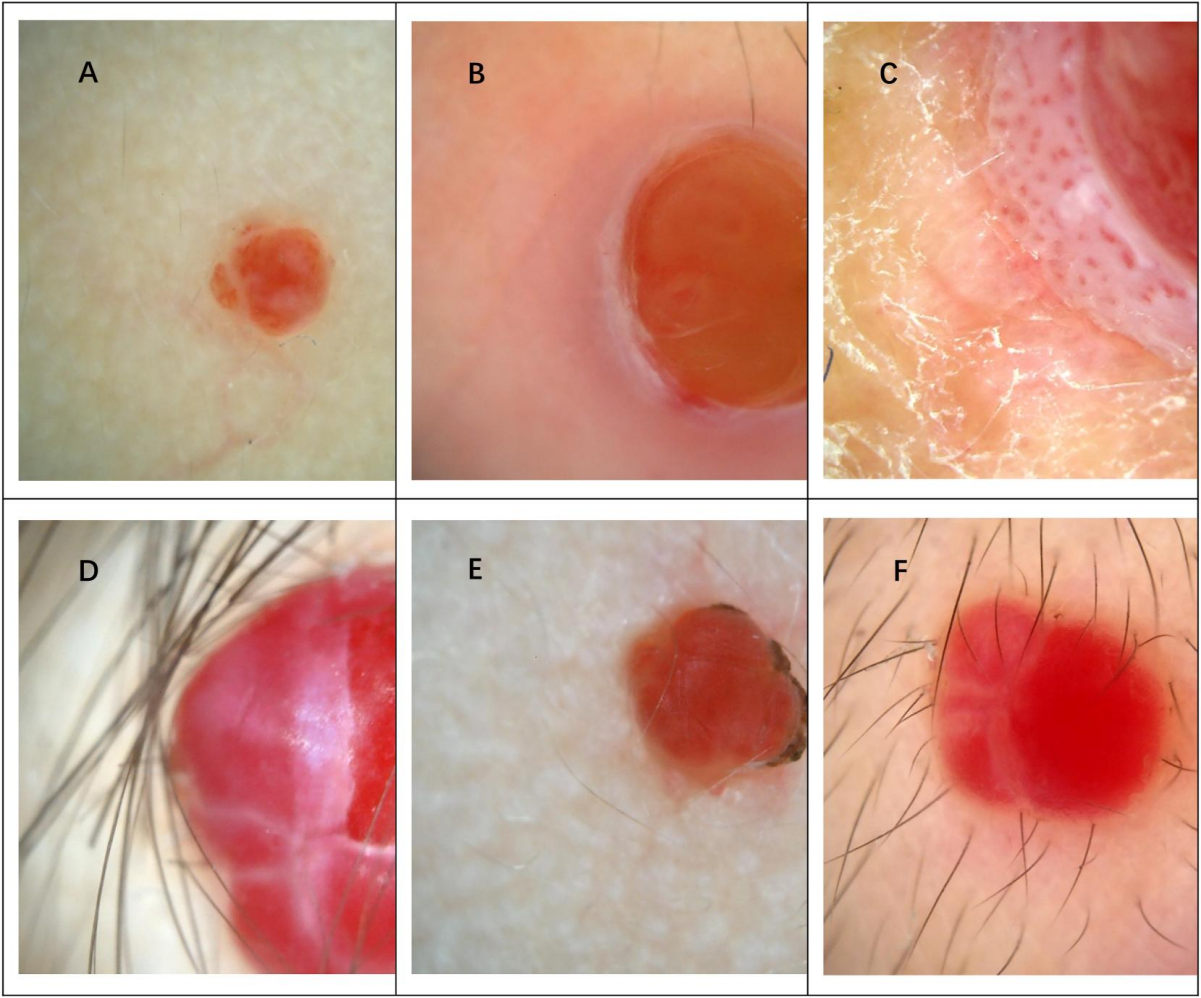
Dermoscopic patterns of pyogenic granuloma of different locations: (A) Face; (B) Face; (C) Trunk; (D) Head; (E) Face; (F) Perilabial area.

**FIGURE 3:**
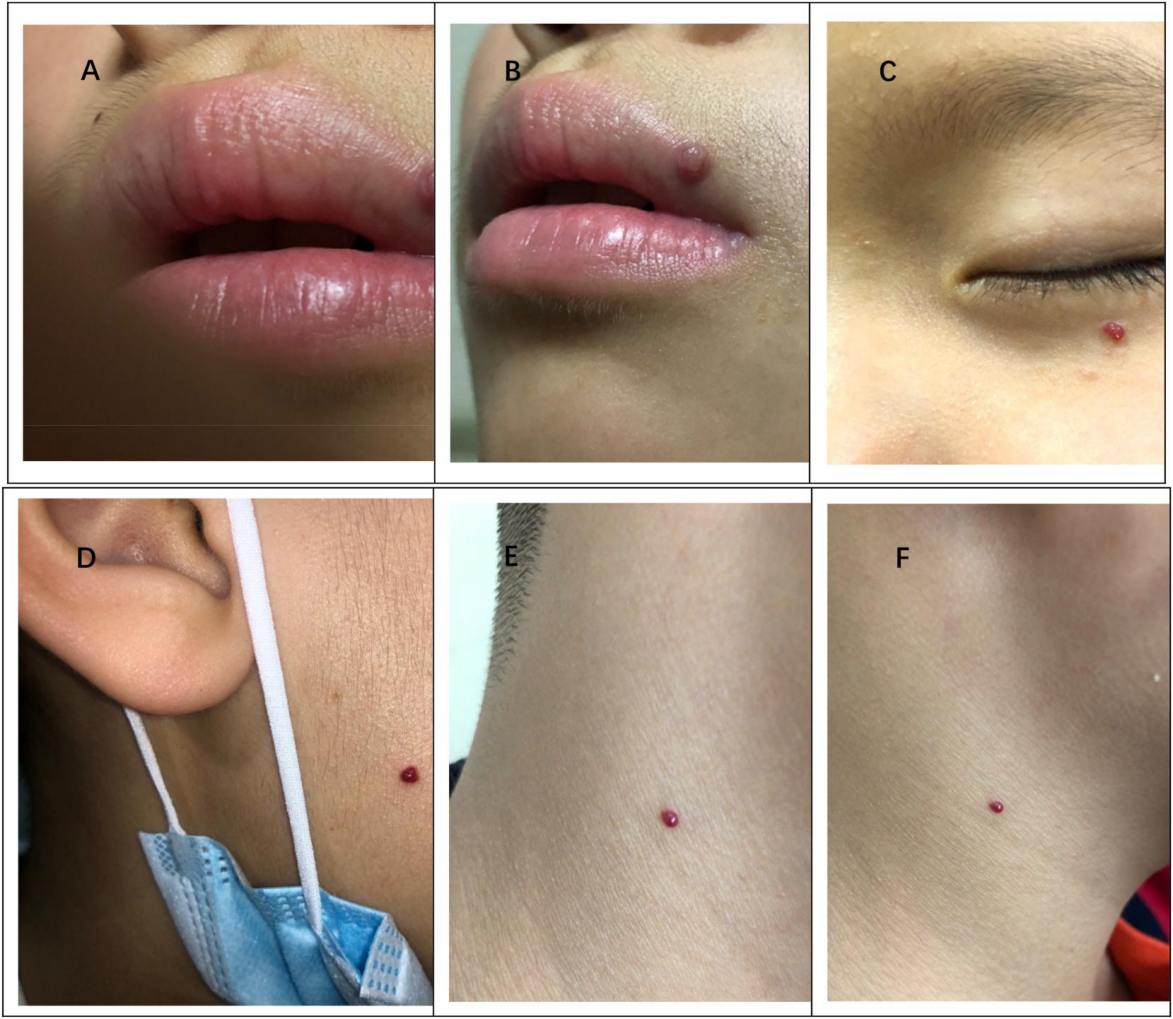
Clinical pictures of different patients with facial pyogenic granulomas.

A total of 36 children with PG were involved, including 14 females (38.89%) and 22 males (61.11%). The median duration of the disease was 1.5 (1.0, 2.25) months, 1 (1, 3) months for females and 2 (1, 2.25) months for males. The distribution of age was basically the same among toddlers, pre‐school children and school‐age students. The average age of onset was mainly focus on pre‐school age, with a median age of 69 (28.75,113) months. The main site of onset was in the face in 26 cases (72.22%) (Tables 1 and 2). The reddish homogeneous areas as dermoscopic pattern were seen in 36 patients (100%), followed by white collarette pattern in 33 cases (91.7%), yellow‐white scales pattern in 25 cases (69.4%), vascular structures pattern in 19 cases (52.8%), and white rail lines pattern in 20 cases (55.6%), Dermoscopic characteristics of hemorrhage or dark red scabs could be seen in a small number of patients (see Figures 1 and 2 for dermatoscopic patterns and Figure 3 for clinical images). There was no statistical difference between gender, age or locations and the dermoscopic patterns (*p* value is greater than 0.05) (Table 3). The multifactor logistic regression equation was constructed by incorporating gender,age,course, location of disease and yellow with white scales as dermoscopic pattern. The results showed that the influence of gender on this dermoscopic pattern was statistically significant (OR = 9.26,95% CI 1.33–64.65, *p* = 0.025). The probability of yellow and white scales in male patients was nearly 8.26 times higher than that in female patients. While there was no statistical difference between the courses or locations and the yellow and white scales pattern (Table 4). When a multifactor logistic regression equation was constructed by incorporating gender, location, age and course of disease groups with white rail lines pattern, the results showed that the effect of course of disease on dermoscopic pattern (white rail line) was statistically significant (OR = 4.62,95% CI 1.05–20.37, *p* = 0.043). That is, the probability of white rail lines pattern in the group with disease course more than 1 month increased by nearly 3.62 times compared with that in the group with disease course less than 1 month. However, there was no statistical difference in the influence of gender, location and age on the white rail lines pattern (Table 5). The multifactor logistic regression equation was constructed by incorporating gender, location, age and course of disease grouping and dark red scabs pattern. The results showed that the influence of gender on dark red scabs pattern was statistically significant (OR = 12.01,95% CI 1.11–130.22, *p* = 0.041), which meant the probability of dark red scabs pattern in men was nearly 11.01 times higher than that in women, while there was no statistical difference in the influence of gender, location and age on dark red scabs pattern (Table 6). A multifactor linear regression equation was constructed by incorporating age, course of disease, sex and location and the number of dermoscopic patterns. The results showed that there was a statistical difference between the influence of different course of disease (months) and the number of dermatoscopic patterns (*b* = 0.22, *t* = 2.27, *p* = 0.030), and there was a statistical difference between gender and the number of dermoscopic patterns (*b* = 0.89, *t* = 2.30, *p* = 0.028). No statistical difference was found between age, location and the number of dermoscopic patterns (*p* = 0.947, *p* = 0.338), suggesting that there was a positive correlation between male and longer course of disease and the number of dermoscopic patterns (Table 7). Looking back at the medical records, only 4 among the involved patients underwent histopathological examination during surgical treatment. The pathological examination results showed intact epidermis, small vessel hyperplasia mainly composed of dermal capillaries, interstitial edema, infiltration of inflammatory cells, and some blood vessels were separated by longitudinal fibrous septa in a lobulated shape, which was consistent with clinical and dermatoscopy manifestations (see supplementary documents for pathological images).

**TABLE 1:** Summary of general data of 36 cases of pyogenic granuloma.

General material		*N* (%)	M (P25, P75)/mean ± SD(95% CI)	
Gender	Female	14 (38.9%)		
	Male	22 (61.1%)		
Location	Non‐facial	10 (27.8)%		
	Facial	26 (72.2%)		
Course^a^	≤1 month	18 (50%)	1 (1, 1)	
	>1 month	18 (50%)	2.5 (2, 3.5)	
Age^b^	≤36 months	11 (30.6%)	20.3 ± 8.1 (14.8–25.7)	69.0 ± 43.5 (54.2–83.7)
	37∼84 months	12 (33.3%)	60.9 ± 16.0 (50.8–71.1)	
	≥85 months	13 (36.1%)	117.6 ± 20.9 (105.0–130.2)	
Atopic history	Yes	19 (52.8%)		
	No	17 (47.2%)		

^a^ The course of disease conforms to a skewed distribution, expressed as “M (P25, P75).”

^b^ Age follows a normal distribution, expressed as “mean ± standard deviation (95% confidence interval).”

**TABLE 2:** Age, course and location distribution of children of different sexes.

General material	Male M (P25, P75)	Female M (P25, P75)	*p*
Age	45.5 (21.3, 86.3)	88.0 (57.8, 119.3)	0.05
Course	2 (1, 2, 3)	1 (1, 3)	0.636
Location	2 (2, 3)	2 (2, 2)	0.123

**TABLE 3:** Summary of dermatoscopic features of 36 cases of Pyogenic Granuloma in children.

Groups	Gender				Age				Location					
Features	Female (*n* = 14)	Male (*n* = 22)	Total	*p*	≤36Mo (3Yrs) (*n* = 11)	37∼84Mo (3∼7Yrs) (*n* = 12)	≥85Mo (>7Yrs) (*n* = 13)	*p*	Head (*n* = 1)	Face (*n* = 26)	Neck (*n* = 4)	Lower limbs (*n* = 3)	Trunk (*n* = 2)	*p*
Reddish or red‐white homogeneous region	14 (100.0%)	22 (100.0%)	36 (100.0%)	——	11 (100.0%)	12 (100.0%)	13 (100.0%)	——	1 (100.0%)	26 (100.0%)	4 (100.0%)	3 (100.0%)	2 (100.0%)	——
Peripheral collarette	13 (92.9%)	20 (90.9%)	33 (91.7%)	1.000	11 (100.0%)	10 (83.3%)	12 (92.3%)	0.629	1 (100.0%)	24 (92.3%)	4 (100.0%)	3 (100.0%)	1 (50.0%)	0.317
Gray white rail lines	9 (64.3%)	11 (50%)	20 (55.6%)	0.501	6 (54.5%)	6 (50%)	8 (61.5%)	0.914	1 (100.0%)	15 (57.7%)	2 (50.0%)	1 (33.3%)	1 (50.0%)	0.952
Large and tortuous vessels in the skin lesion or at the edge	6 (42.9%)	13 (59.1%)	19 (52.8%)	0.495	6 (54.5%)	7 (58.3%)	6 (46.2%)	0.915	0 (0.0%)	12 (46.2%)	3 (75.0%)	2 (66.7%)	2 (100.0%)	0.452
Hemorrage	1 (7.1%)	9 (40.9%)	10 (27.8%)	0.054	5 (45.5%)	2 (16.7%)	3 (23.1%)	0.331	0 (0.0%)	5 (19.2%)	3 (75.0%)	1 (33.3%)	1 (50.0%)	0.102
White or yellow scales	7 (50.0%)	18 (81.8%)	25 (69.4%)	0.067	7 (63.6%)	9 (75.0%)	9 (69.2%)	0.903	1 (100.0%)	17 (65.4%)	3 (75.0%)	3 (100.0%)	1 (50.0%)	0.828
Dark red scab	1 (7.1%)	9 (40.9%)	10 (27.8%)	0.054	5 (45.5%)	2 (16.7%)	3 (23.1%)	0.331	0 (0.0%)	6 (23.1%)	2 (50.0%)	1 (33.3%)	1 (50.0%)	0.609

*Note*: Data information: The data are presented as Fisher's precision probability test since the sample was smaller than 40.

**TABLE 4:** Results of correlation analysis between gender, age, course of disease, location and PG dermatoscopic features (yellow and white scales).

Variations	*b* value	SE value	Wald value	*p* Value	OR value	95% CI of OR
Constant	−2.54	1.92	1.76	0.184	0.08	
Gender^a^	2.23	0.99	5.04	0.025	9.26	1.33–64.65
Section	−0.22	0.49	0.20	0.654	0.80	0.31–2.10
Age	0.01	0.01	1.72	0.190	1.01	0.99–1.04
Course	−0.23	0.24	0.92	0.337	0.80	0.50–1.27

^a^ Compared to female.

**TABLE 5:** Results of correlation analysis between gender, location, age, disease course groups and dermoscopic features (gray and white rail lines in the lesions).

Variations	*b* value	SE value	Wald value	*p* Value	OR value	95% CI of OR
Constants	−0.26	2.35	0.01	0.913	0.77	
Gender^a^	−0.79	0.82	0.92	0.337	0.45	0.09–2.28
Location	−0.20	0.43	0.20	0.652	0.82	0.35–1.92
Ages^b^	−0.02	0.80	0.00	0.981	0.98	0.20–4.75
Disease course^c^	1.53	0.76	4.10	0.043	4.62	1.05–20.37

^a^ Compared to female.

^b^ The age group is divided into ≤36 months and>36 months old groups, and the less than 36 months old group is the control group.

^c^ The course of disease is divided into ≤1 month and>1 month groups, and ≤1 month group is the control group.

**TABLE 6:** Results of correlation analysis between gender, location, age and course of disease groups and the characteristics of PG dermoscopy (dark red scab).

Variations	*b* value	SE value	Wald value	*p* Value	OR value	95% CI of OR
Constants	−3.41	2.64	1.67	0.197	0.03	
Gender^a^	2.49	1.22	4.18	0.041	12.01	1.11–130.22
Location	0.21	0.45	0.21	0.645	1.23	0.51–2.94
Age	0.00	0.01	0.00	0.951	1.00	0.98–1.02
Course of disease^b^	−1.60	0.91	3.14	0.076	0.20	0.03–1.19

^a^ Female group as control group.

^b^ The course of disease was divided into ≤1 month and>1 month groups, with<1 month group as control group.

**TABLE 7:** The results of multivariate linear regression analysis of age, course of disease, sex, location and dermatoscopic features.

Variations	*b* value	SE value	Standardized value of *B* value	*t* value	*p* Value
Constants	1.84	0.80		2.31	0.028
Age	0.00	0.00	0.01	0.07	0.947
Course of disease	0.22	0.10	0.35	2.27	0.030
Gender^a^	0.89	0.39	0.37	2.30	0.028
Location	0.19	0.20	0.15	0.97	0.338

^a^ Female group as control group.

## 4 DISCUSSION

It is reported that the incidence rate of PG accounts for 3.81%–7% of the pathological examination results of oral mucosal lesions.^11^ In childhood, the prevalence of malignant skin tumors with similar manifestations to PG is low. Catherine H L Hong et al found that most of the biopsies of oral mucosal lesions in children from 1 January 1990 to 31 December 2018 were benign, of which PG is mostly common seen.^12^ Since PG is prone to occur in the face in children, scarring after pathological examination will cause possible disfiguring problems. Taking consider that preschool‐age children cooperate poorly during invasive examination, non‐invasive dermatoscopy is more suitable for diagnosis and evaluation of the disease. Pyogenic granuloma has multiple patterns under dermatoscopy, and the specificity would reach 100% with the appearance of reddish homogeneous structure, white collarette and white rail lines together.^13^ In our study, 16 patients (50%) had the mentioned three dermatoscopic patterns at the same time, and 33 patients (92%) had the reddish homogeneous structure and white collarette sign at the same time. It is speculated that the presence of both a reddish homogeneous area and a white collarette sign is a highly sensitive indicator of PG in children patients. Childhood PG needs to be differentiated from non‐pigmented melanoma, Spitz nevus, infantile hemangioma, etc. These diseases have different characteristics under dermatoscopy. Both amelanotic Melanoma and PG can show reddish background under dermatoscopy, but melanoma shows a variety of different shades of pink, and there are significant central blood vessels, and the dotted, hair‐pin like and linear irregular blood vessels would appear at the same time. Also, other manifestations such as blue‐white screen, scar like depigmentation, multiple blue gray dots and irregular brown spots/globular structure could be seen.^14^ Pink homogeneous pattern can also be seen under dermatoscopy in Spitz nevus, but with regular distribution of dotted blood vessels under a pink background.^15^ Infantile hemangiomas can be seen under dermatoscopy with twisted or spherical blood vessels converging into multiple blocky lacunar structures under a red background.^10^

The reddish homogeneous areas could be seen in all patients in the study. The pathological feature may be a large number of small capillaries or proliferative vessels in the mucus matrix,^16^ which is consistent with previous studies,^17^, ^18^ suggesting that this pattern is still one of the main characteristic evaluation indicators in patients with PG at early stage. This study showed that the portion of patients with yellow‐white scales as dermoscopic pattern was 69.4%, while 27.8% showed dark red scabs and bleeding under the dermatocope, which was higher than that in previous studies,^8^, ^19^supposing that these three features are specific to distinguish pediatric PG from elder patients (>14 years old).In our study, 52.8% of the patients had a history of atopic diseases (clearly diagnosed by a specialist with allergic rhinitis, asthma, or eczema/atopic dermatitis). And yellow‐white scales were seen in 68.4% of these patients who had atopic history on their scalps and lower extremities, suggesting that yellow‐white scales may be related to the drier skin on scalp and lower extremities, or related to the atopic constitution and the destruction of skin barrier function of the patients.^20^ According to the report of Pagliai KA, 8% of children with PG have complications such as atopic dermatitis,^21^ which can increase the risk of itching or trauma. Since immunity and inflammation are common causes of PG, we suspect that children with dry skin or atopic constitution are more likely to having PG. However, whether this feature is closely related to the cause of PG still needs further prospective researches to verify. In addition, skin lesions with surface scales can also occur in angiokeratomas and other diseases,^22^ which will increase the probability of misdiagnosis. Thus, we should be more careful when we reach the diagnosis of PG especially in scalp and lower limbs.

Our study discovered that there was no statistical difference between the gender, age and locations of the patients and the different dermoscopic patterns of PG. It suggested that pediatric patients could have various typical dermoscopic manifestations of PG regardless of gender, location and age stage, proving that dermoscopic examination could be an effective diagnosis method for children with PG at early stage. Pagliai KA and others have reported a study involving 128 children with PG, that the average age was 5.9 years old, male patients accounted for 60.7%, patients with PG on head or neck accounted for 76.9%.^21^ Our study revealed that PG was more common in male pediatric patients at preschool age with face as location and the duration of the disease less than 1 month, which was consistent with previous studies.^22^ Considering that male children and preschool children are more active, exploratory with poorer self‐discipline and poor sense of self‐protection, higher probability of trauma accident would happen to them. Hence, PG is in favor of this above population. Meanwhile, the parents of this population usually pay more attention to their children's health condition because of their age, so it is easier to find out lesions at early stage, which makes the course of disease is relatively short.

In our study, yellow‐white scales and dark red scabs as dermoscopic patterns were more likely to be found in male children than in female. The fact that boys are more likely to scratch and lead to trauma or that boys usually have drier skin might explain. Besides of common patterns as reddish homogeneous areas, yellow‐white scales and dark red scabs as dermoscopic patterns should be paid more attention to when conducting dermoscopic examination for male children with PG. In our study, the pattern of white rail lines was more often observed in patients with longer course of disease (longer than 1 month), which may correspond histologically to the fibrous septa that surround the capillary tufts or lobules.^23^ The pattern is actually consistent with the pathological evolution of the disease. That's why it is hardly to see the typical white rail lines in the skin lesions under dermatoscopy in patients with shorter course of disease (less than or equal to 1 month). As a result, clinicians should keep other more common dermoscopic patterns of PG (such as reddish homogeneous areas, white collarette, etc.) in mind to avoid missed diagnosis or misdiagnosis. Another surprising finding was that male patients had more dermoscopic patterns than female patients and patients with a longer course of disease (more than 1 month). More typical dermoscopic patterns were also observed in male patients, suggesting that female children patients with a shorter course of disease would have relatively fewer and less typical dermoscopic patterns, which makes the diagnosis more difficult. Physicians should give more careful examination to this population. Then other assistant examinations should be considered when necessary.

In conclusion, our study exhibited similar dermoscopic patterns in childhood PG at early age than adulthood. As a simple and non‐invasive tool, dermoscopy could be used more frequently in the evaluation in children with PG at early stage for its economic efficiency and practicability. However, our study, as a retrospective analysis, is still limited to its small sample size and its selective bias to pediatric populations. There is selective bias and a lack of histopathological support for part of the patients involved. Therefore, this study could only provide referential information for clinical practice. On the other hand, this study could also inspire more prospective researches of high‐quality with large samples in the future to better analyze and discover the characteristics of PG, so as to improve the efficiency of diagnosis and treatment and to serve patients better by reducing the probability of invasive examination.

## AUTHOR CONTRIBUTIONS

Conceptualization, Shijuan Yu, Xiaoyan Luo, Hua Wang and Jingyi He; Methodology, Li Wang and Qi Tan; Investigation, Shijuan Yu, Li Wang and Qi Tan; Formal Analysis, Shijuan Yu, Hua Wang, Xiaoyan Luo and Jingyi He; Writing‐Original Draft, Shijuan Yu and Jingyi He; Writing‐Review & Editing, Shijuan Yu, Hua Wang and Jingyi He; Resources, Hua Wang and Xiaoyan Luo; Supervision, Jingyi He.

## CONFLICT OF INTEREST STATEMENT

None of the authors have a conflict of interest to disclose in this manuscript.

## ETHICS STATEMENT

All procedures in the study involving human participants were in accordance with the ethical standards of the institutional and national research committee. This study was approved by the Institutional Review Board, Children's Hospital of Chongqing Medical University, Chongqing, China (No. 2022‐567). The informed contents for the research and publication were obtained from the patients' parents.

## Supporting information

Figure S1

Figure S2

Figure S3

Figure S4

Figure S5

Figure S6

Figure S7

Figure S8

Figure S9

Figure S10

Figure S11

## Data Availability

The data that support the findings of this study are available from the corresponding author upon reasonable request.
