# Supplementary figures and images for: Dermatoscopic features of pyogenic granuloma in children
Source: Pediatr Discov. 2024 Jun 17;2(2):e73. doi: 10.1002/pdi3.73 (PMC12118305; doi:10.1002/pdi3.73)

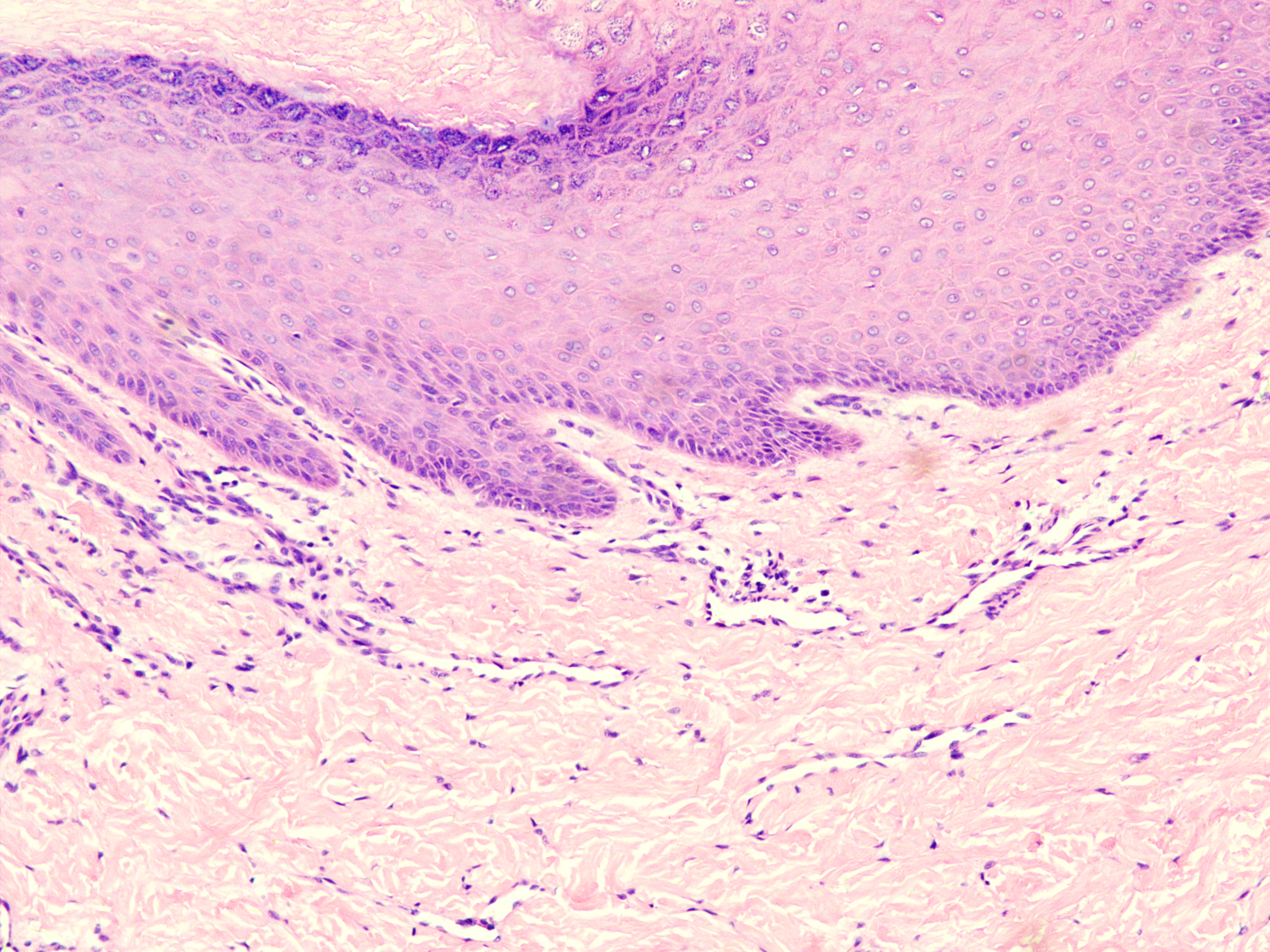

Supplement: Supplementary file 1 — Figure S1 [file PDI3-2-e73-s005.jpg]

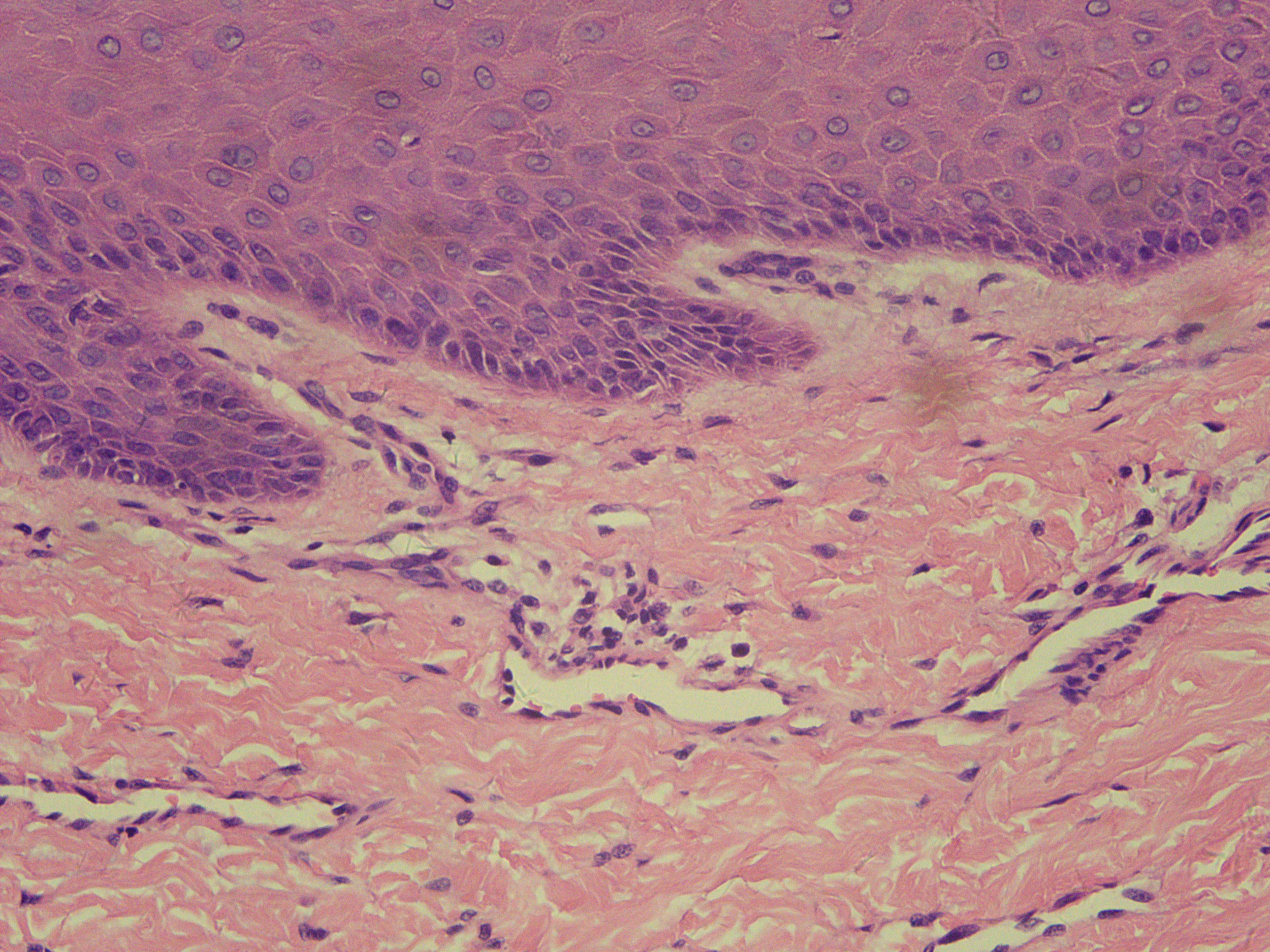

Supplement: Supplementary file 2 — Figure S2 [file PDI3-2-e73-s008.jpg]

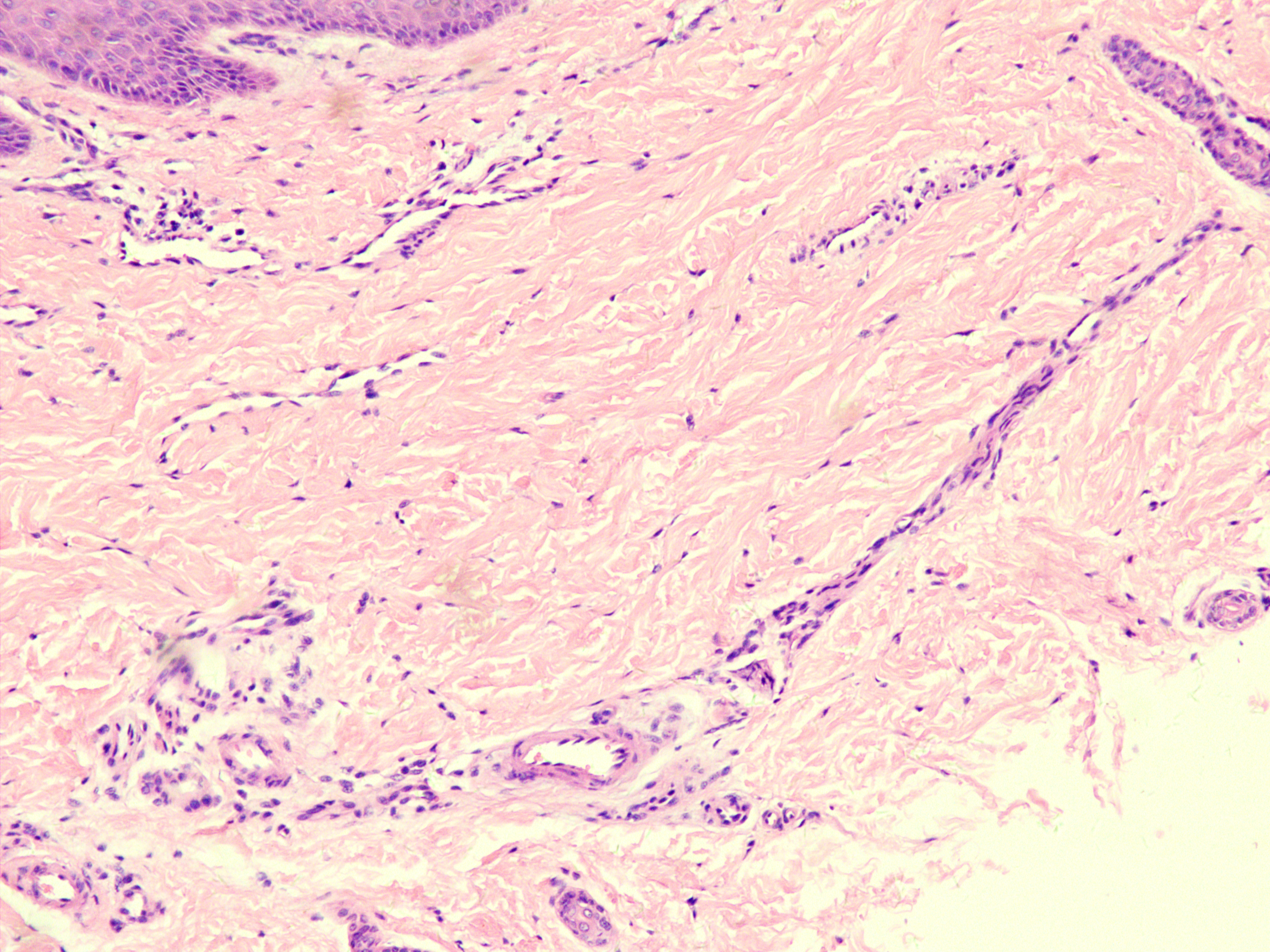

Supplement: Supplementary file 3 — Figure S3 [file PDI3-2-e73-s009.jpg]

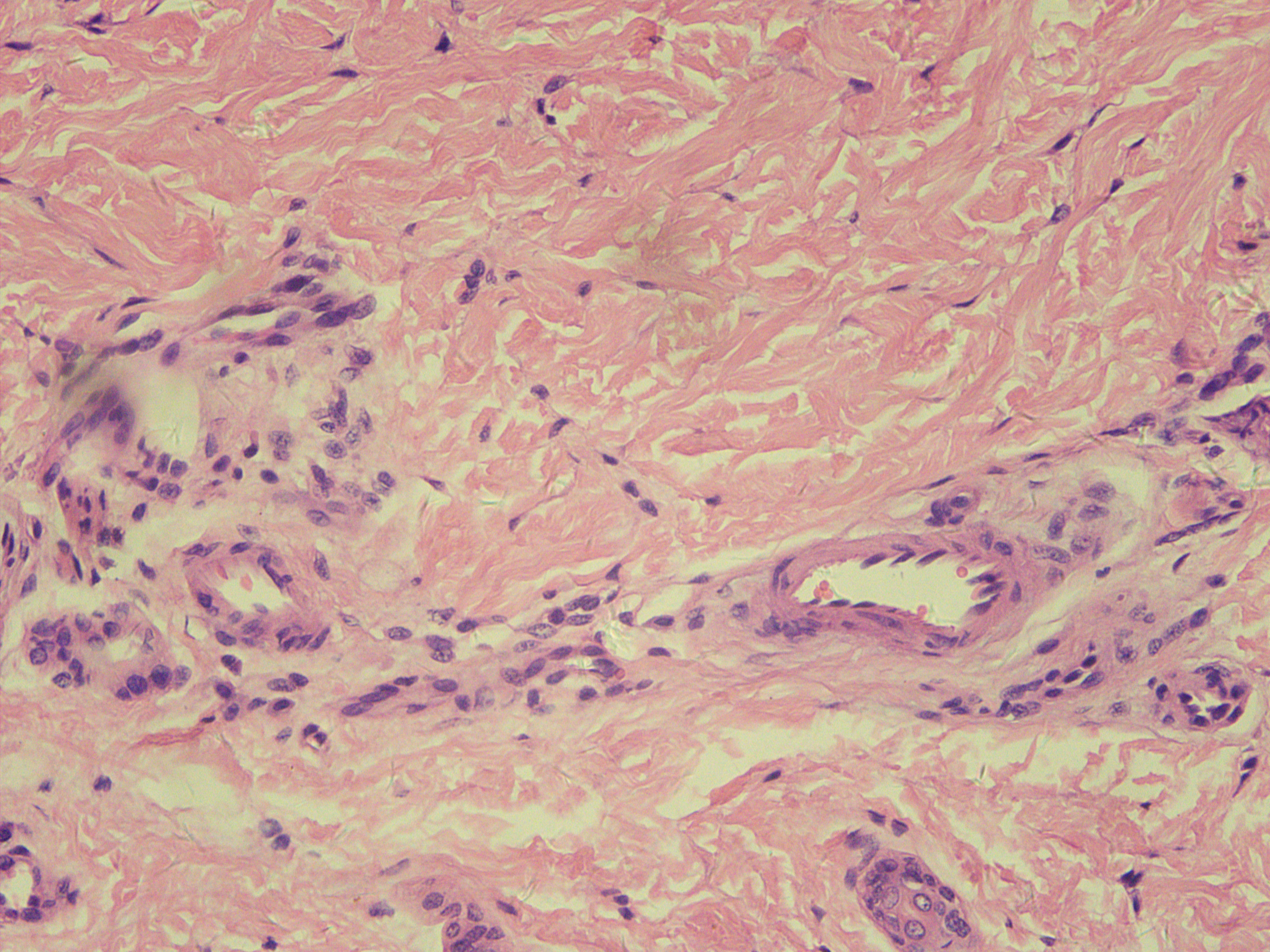

Supplement: Supplementary file 4 — Figure S4 [file PDI3-2-e73-s011.jpg]

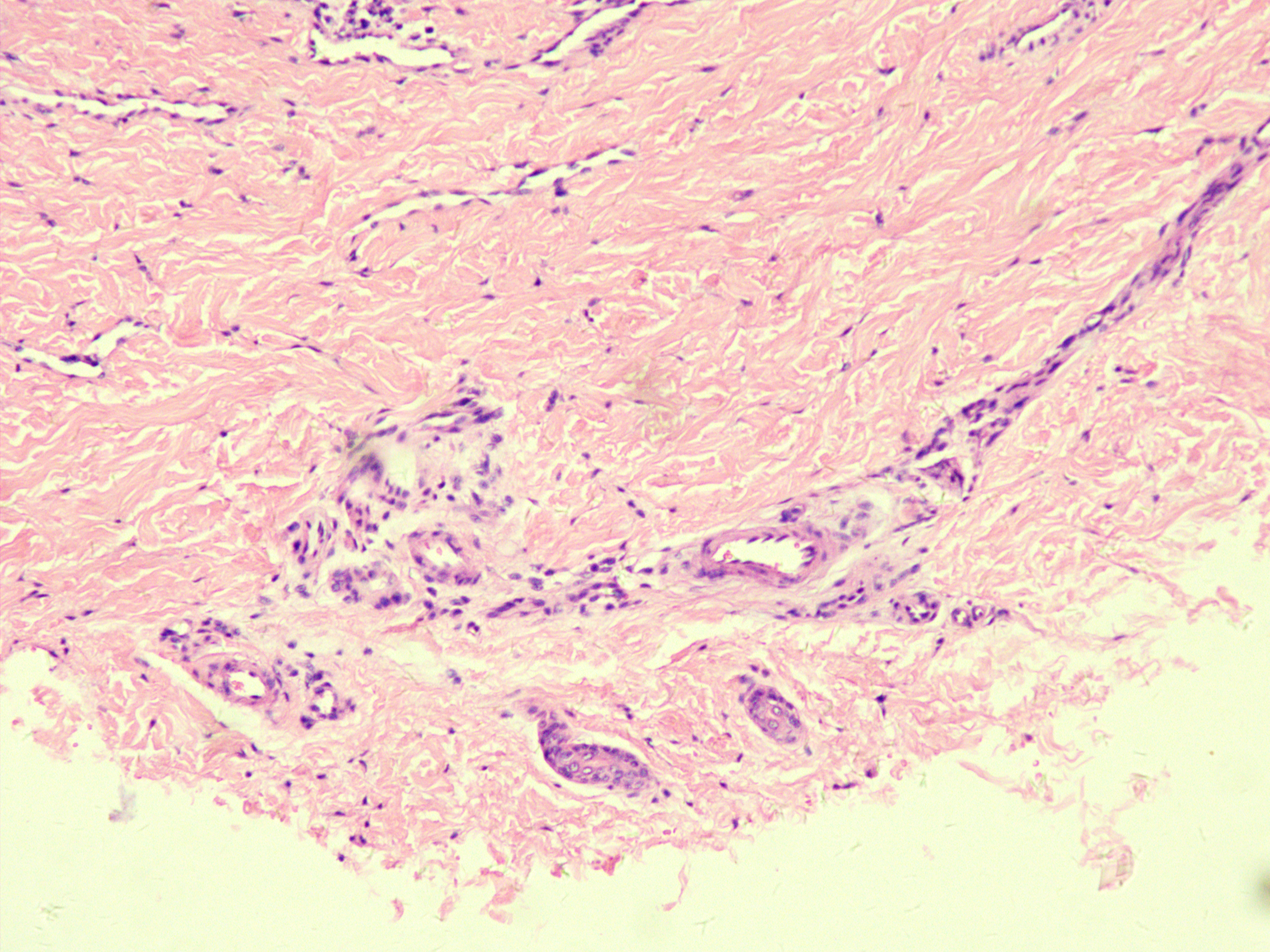

Supplement: Supplementary file 5 — Figure S5 [file PDI3-2-e73-s002.jpg]

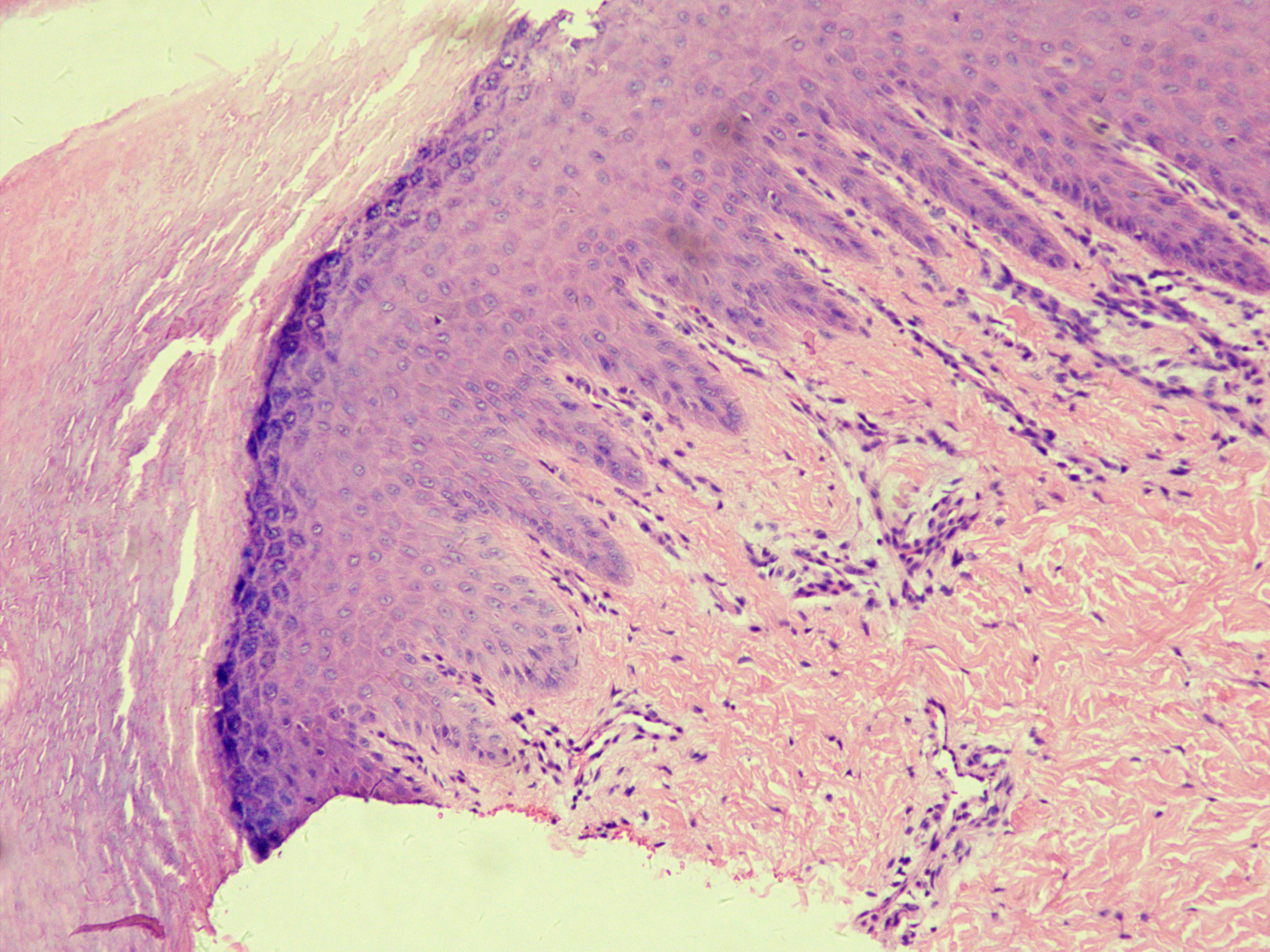

Supplement: Supplementary file 6 — Figure S6 [file PDI3-2-e73-s003.jpg]

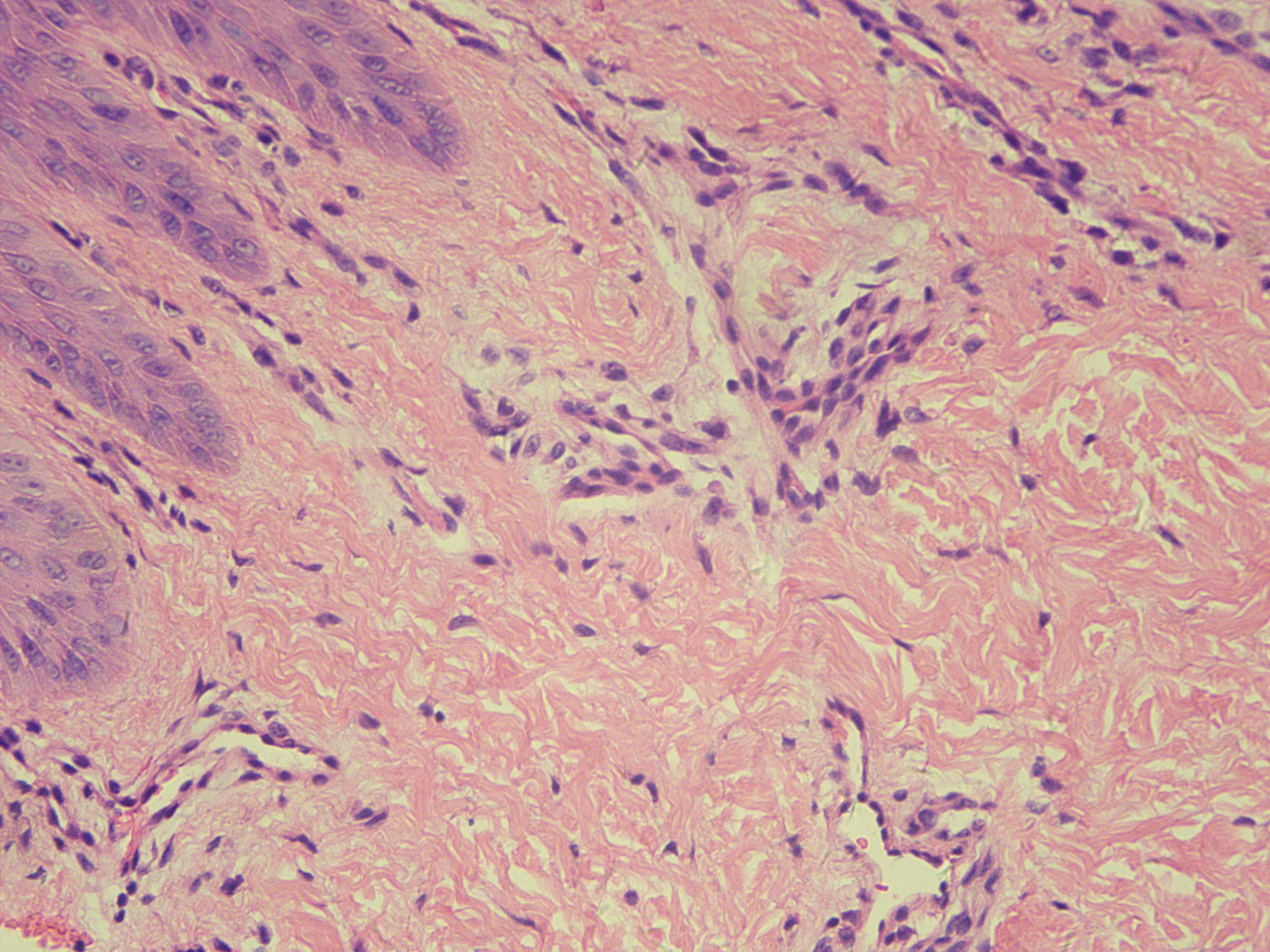

Supplement: Supplementary file 7 — Figure S7 [file PDI3-2-e73-s010.jpg]

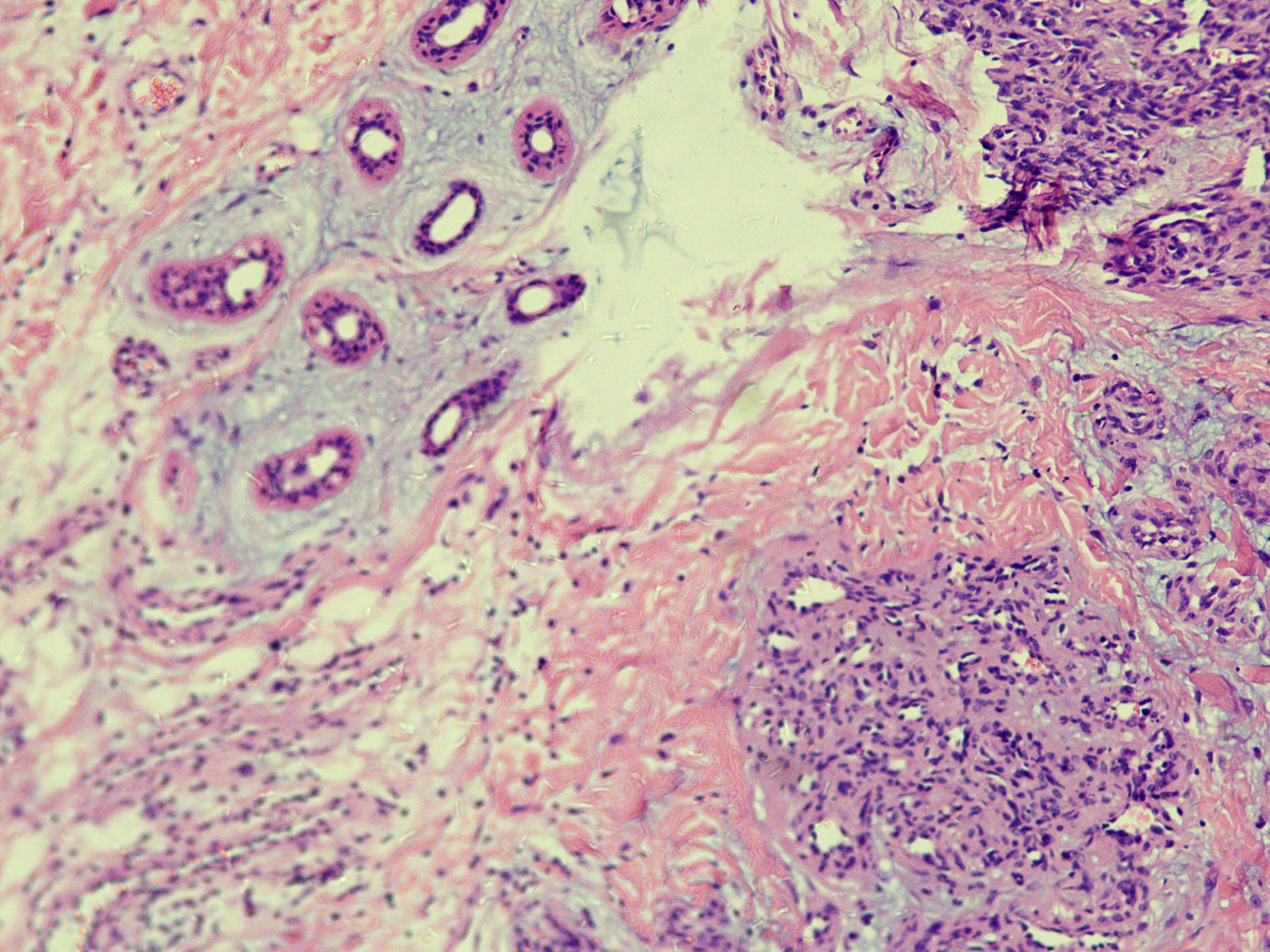

Supplement: Supplementary file 8 — Figure S8 [file PDI3-2-e73-s004.jpg]

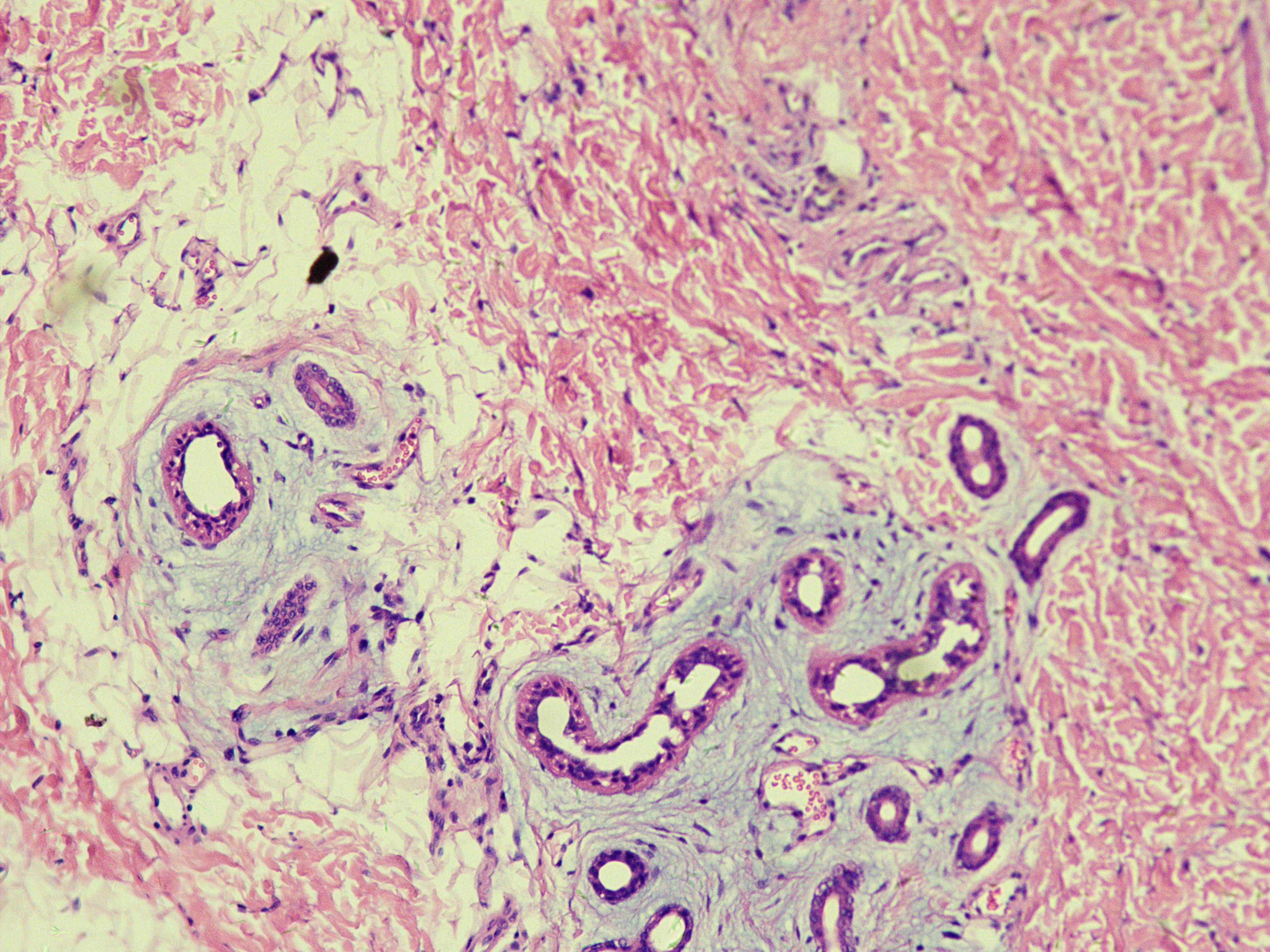

Supplement: Supplementary file 9 — Figure S9 [file PDI3-2-e73-s007.jpg]

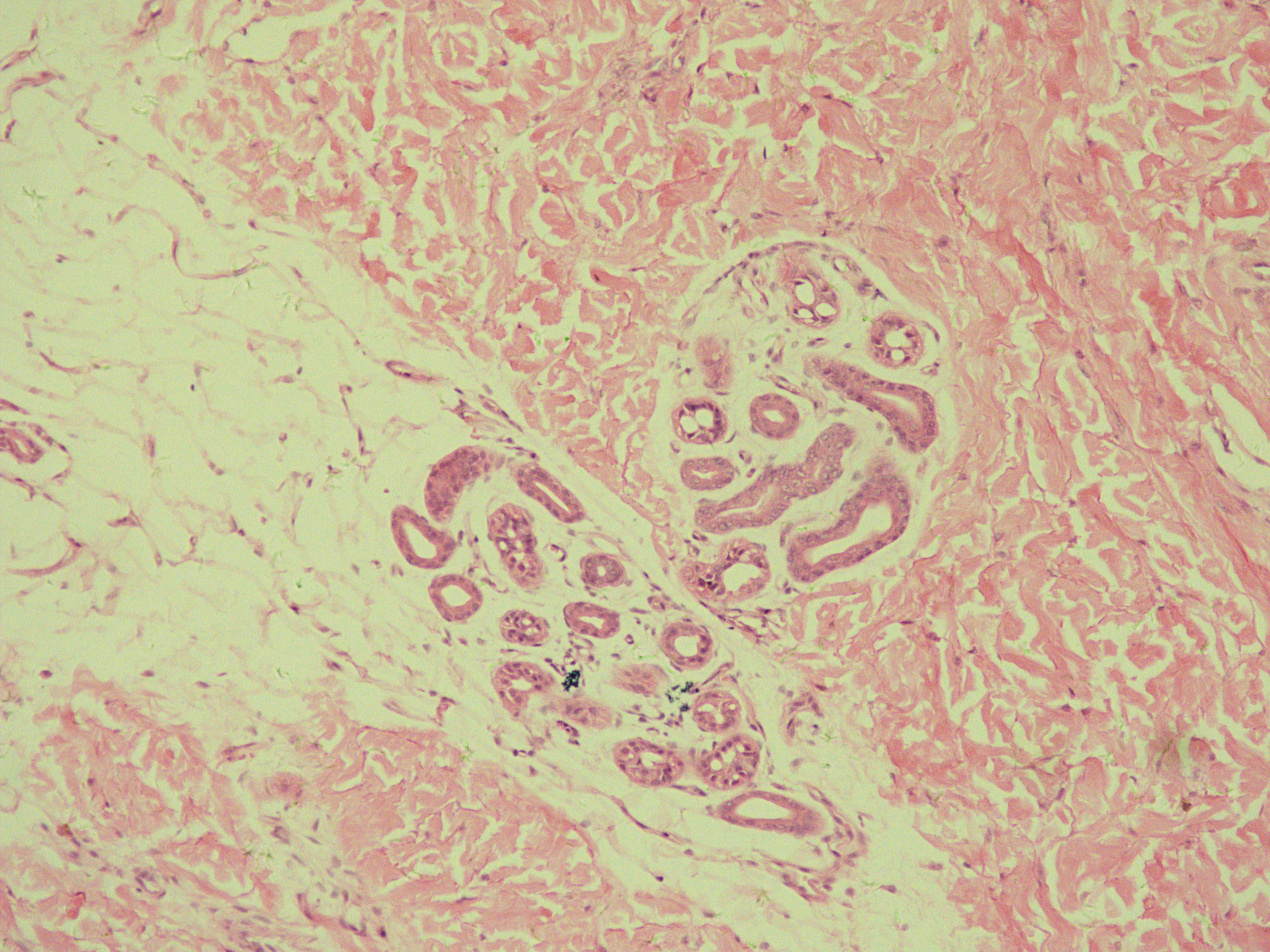

Supplement: Supplementary file 10 — Figure S10 [file PDI3-2-e73-s001.jpg]

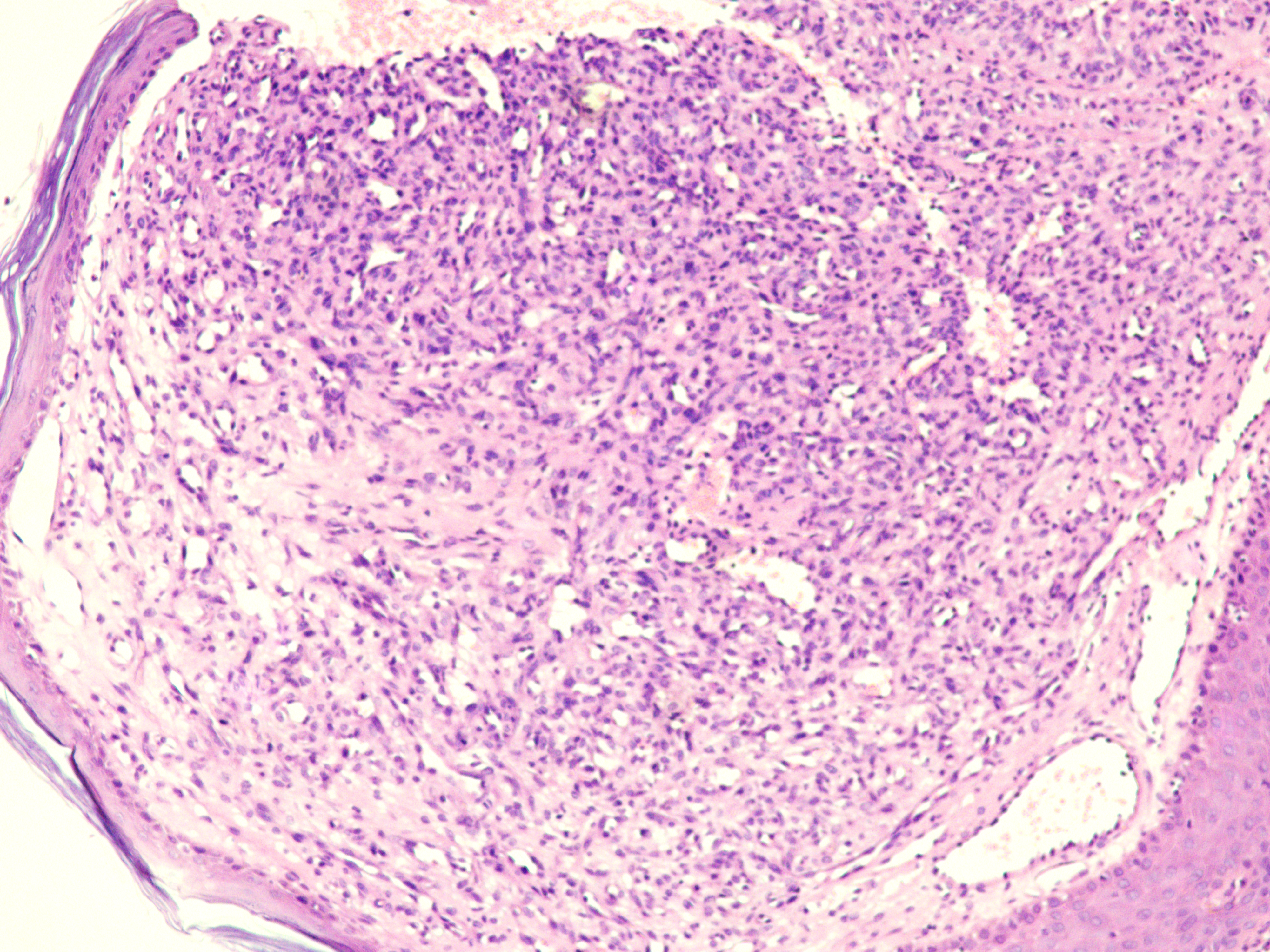

Supplement: Supplementary file 11 — Figure S11 [file PDI3-2-e73-s006.jpg]
